# A robust confidence–accuracy dissociation via criterion attraction

**DOI:** 10.1093/nc/niab039

**Published:** 2021-11-15

**Authors:** Dobromir Rahnev

**Affiliations:** School of Psychology, Georgia Institute of Technology, 654 Cherry Str. NW, Atlanta, GA 30332, USA

**Keywords:** criterion attraction, confidence, metacognition, perceptual decision making

## Abstract

Many studies have shown that confidence and accuracy can be dissociated in a variety of tasks. However, most of these dissociations involve small effect sizes, occur only in a subset of participants, and include a reaction time (RT) confound. Here, I develop a new method for inducing confidence–accuracy dissociations that overcomes these limitations. The method uses an external noise manipulation and relies on the phenomenon of criterion attraction where criteria for different tasks become attracted to each other. Subjects judged the identity of stimuli generated with either low or high external noise. The results showed that the two conditions were matched on accuracy and RT but produced a large difference in confidence (effect appeared for 25 of 26 participants, effect size: Cohen’s *d* = 1.9). Computational modeling confirmed that these results are consistent with a mechanism of criterion attraction. These findings establish a new method for creating conditions with large differences in confidence without differences in accuracy or RT. Unlike many previous studies, however, the current method does not lead to differences in subjective experience and instead produces robust confidence–accuracy dissociations by exploiting limitations in post-perceptual, cognitive processes.

**Highlights**

- A new method is developed for inducing confidence–accuracy dissociations using external noise and criterion attraction.
- The method leads to large effects and consistent results across participants.
- The novel confidence–accuracy dissociation includes no reaction time confounds.
- Results point toward criterion attraction as a robust effect that is likely to have wide applicability.

## Introduction

It is well known that across a variety of tasks, subjective ratings of confidence tend to closely track one’s objective level of performance (Mamassian 2016). The close correspondence between confidence and accuracy has made studying subjective evaluation especially challenging because it has been difficult to separate it from objective performance on the main task (Morales *et al.* 2015a, 2019). One strategy for understanding the factors that specifically drive confidence has been to create conditions that are matched in objective performance (i.e. stimulus sensitivity) but differ in subjective performance (i.e. confidence).

### Confidence–accuracy dissociations

The last decade has seen a proliferation of experiments on how subjective and objective performance can be dissociated. Most of this work has been done in the domain of perception. This research program has demonstrated that confidence–accuracy dissociations can be produced by a number of different factors including positive evidence bias (Zylberberg *et al.* 2012; Koizumi *et al.* 2015; Maniscalco *et al.* 2016; Samaha *et al.* 2016; Peters *et al.* 2017; Odegaard *et al.* 2018), stimulus variability (Zylberberg *et al.* 2014, 2016; de Gardelle and Mamassian 2015; Spence *et al.* 2016, 2018; Boldt *et al.* 2017, 2019; Desender *et al.* 2018), motor preparation and execution (Fleming *et al.* 2015; Gajdos *et al.* 2019), visual field location (Solovey *et al.* 2015; Li *et al.* 2018), transcranial magnetic stimulation (Rounis *et al.* 2010; Rahnev *et al.* 2012b, 2016; Shekhar and Rahnev 2018), varying pre-stimulus brain activity (Rahnev *et al.* 2012a; Samaha *et al.* 2017), confidence on the previous trial (Rahnev *et al.* 2015; Aguilar-Lleyda *et al.* 2021), attention (Wilimzig *et al.* 2008; Rahnev *et al.* 2011; Kurtz *et al.* 2017; Recht *et al.* 2019), arousal (Allen *et al.* 2016), unconsciously presented information (Vlassova *et al.* 2014), and stimulus visibility (Rausch *et al.* 2018). Many other factors that cause such dissociations are likely to be discovered in the coming years (Rahnev *et al.* 2021).

However, despite the existence of a very large number of confidence–accuracy dissociations, most previous manipulations are limited in three different ways. First, the dissociations are typically small in magnitude. For example, if confidence is collected on a 4-point scale for two conditions matched on accuracy, most manipulations result in a confidence difference of about 0.1 and virtually never exceed 0.2. Second, many of the dissociations appear in some but not all participants. Third, while this is rarely explicitly reported, many dissociations include a reaction time (RT) confound such that the conditions that differ in confidence are matched in accuracy but not in RT. Indeed, when RT has been reported, it has often been shorter in the condition with high confidence (Samaha *et al.* 2016; Boldt *et al.* 2019). Such RT effects can even lead to questions as to whether these dissociations truly show a divergence of subjective confidence from objective performance because, in many theories, RTs are an integral part of the signal on which confidence ratings are based (Hanks *et al.* 2011; Fetsch *et al.* 2014a,b; Zylberberg *et al.* 2016).

While not every manipulation suffers from all three limitations, it is currently unclear whether any method induces confidence–accuracy dissociations that are large in magnitude, consistent across participants, and free of RT confounds. Given the importance of robustly dissociating confidence and accuracy for understanding the neural and computational mechanisms of confidence (Shekhar and Rahnev 2021a), it is important to exactly develop such manipulations.

### Employing criterion attraction in an external noise paradigm

Here I explore whether a robust confidence–accuracy dissociation can be induced by exploiting the principle of criterion attraction and combining it with an external noise paradigm. Criterion attraction is the idea that when people perform two interleaved tasks that optimally require different criteria, the actual criteria used for the two tasks become ‘attracted’ toward each other. The phenomenon was first demonstrated by Gorea and Sagi in a series of experiments that suggested that in some conditions the criteria may even collapse onto the same unified criterion (Gorea and Sagi 2000, 2001, 2002). Whether the criterion is truly unified across conditions has been a source of controversy (Kontsevich *et al.* 2002; Denison *et al.* 2018; Lee *et al.* 2021) but criterion attraction can be a robust phenomenon even if the criteria from the different conditions never completely collapse onto each other.

It has been argued that to convincingly establish the location of one’s internal criterion, it is necessary to employ an external noise paradigm (Denison *et al.* 2018; Lee *et al.* 2021). In external noise paradigms, the stimulus values of the to-be-discriminated categories are themselves sampled from a distribution (Dosher and Lu 1998; Gold *et al.* 2004; Lu and Dosher 2008; Qamar *et al.* 2013; Cabrera *et al.* 2015). The use of external noise in perceptual paradigms has a long history (Nagaraja 1964; Burgess *et al.* 1981; Legge *et al.* 1987) with its main advantage being that it makes it possible for the internal noise in the visual system to be benchmarked against an externally measurable quantity, which also often leads to more robust results that are consistent across observers (Lu and Dosher 2014).

In fact, Zak *et al.* (2012) used external noise to study criterion attraction. They demonstrated that for some participants the decision criteria across two conditions collapsed, although for other participants the criteria only became attracted to each other without becoming the same. The Zak *et al*.’s study thus demonstrates that criterion attraction can be studied using an external noise paradigm.

However, the Zak *et al.* (2012) study and previous research by Gorea and Sagi focused exclusively on criterion attraction for the decision criterion, which separates the main choice between the two stimulus categories. The principle of criterion attraction (mostly in its extreme form of complete criterion collapse) has been applied to confidence criteria in several studies to explain findings of confidence–accuracy dissociations (Rahnev *et al.* 2011, 2012a,b; Solovey *et al.* 2015; Morales *et al.* 2015b; Li *et al.* 2018), but these studies did not use external noise paradigms.

### The current study

**Figure 1.**
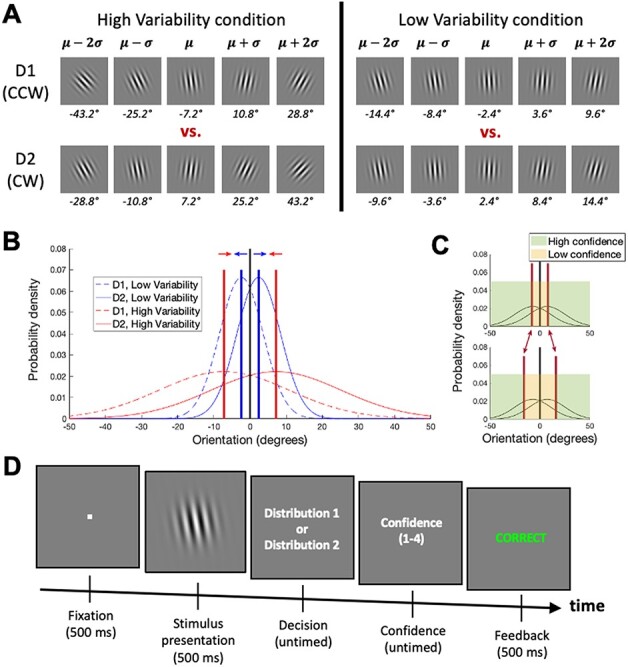
Experimental paradigm. (A) Participants judged the distribution (D1 vs. D2) that generated a stimulus with a given orientation. D1 was biased toward CCW orientations, while D2 was biased toward CW orientations. In the high variability condition, D1 and D2 had high means ($\mu = \pm 7.2^\circ $) and SDs ($\sigma = 18^\circ $), whereas in the low variability condition, D1 and D2 had low means ($\mu = \pm 2.4^\circ $) and SDs ($\sigma = 6^\circ $). The means and SDs in the high variability condition were thus exactly three times higher than in the low variability condition, resulting in identical maximum performance of $d{^{^{\prime}}} = 0.8$ in both cases. For each distribution, the figure displays the orientations corresponding to $\mu - 2\sigma $, $\mu - \sigma $, $\mu $, $\mu + \sigma $, and $\mu + 2\sigma $. CCW (CW) orientations are indicated with negative (positive) numbers. (B) The two sets of distributions, together with the locations of confidence criteria that result in equivalent confidence ratings in the two conditions. If the criteria in the two conditions become ‘attracted’ to each other (see arrows), then the confidence criteria would become conservative for the low variability condition (resulting in low confidence) and liberal for the high variability condition (resulting in high confidence). (C) Depiction of a criterion shift. An outward move of the criteria (transition from top to bottom graph, corresponding to the expected shift for the low variability condition) results in a smaller area of high confidence. Conversely, an inward movement of the criteria (transition from bottom to top graph, corresponding to the expected shift for the high variability condition) results in a larger area of high confidence. (D) Task. On each trial, participants indicated the likely generating distribution and gave a confidence rating on a 4-point scale. Trial-by-trial feedback was provided throughout the whole experiment. The low and high variability distributions were presented in clearly marked, alternating blocks of 50 trials each

Here I specifically examine attraction for confidence criteria in an external noise paradigm to explore whether this approach can lead to a more robust confidence–accuracy dissociation. Full-contrast Gabor patches were presented with orientations sampled from two overlapping distributions. Participants judged which distribution was more likely to have generated each stimulus and provided a confidence rating. Critically, I included two different experimental conditions: in the low variability condition, the two distributions had similar means and low standard deviations (SDs), whereas in the high variability condition, the distributions had dissimilar means and high SDs (Fig. 1A). The means and SDs in the two conditions were proportionate so that the tasks were equally difficult (Fig. 1B). Furthermore, the optimal decision criterion for both conditions was identical [simply judge whether orientation is clockwise (CW) or counterclockwise (CCW) from vertical orientation]. However, in the presence of criterion attraction, the confidence criteria would move outward in the low variability condition (resulting in lower confidence) and inward in the high variability condition (resulting in higher confidence; Fig. 1C).

To anticipate, I found that both stimulus sensitivity (*d*’) and RT were matched across the low and high variability conditions. However, there was a robust confidence dissociation with the low variability condition resulting in lower confidence for 25 of the 26 participants compared to the high variability condition. Estimation of the criterion locations for each condition and additional computational modeling suggested that this effect is consistent with a mechanism of criterion attraction.

## Methods

### Participants

Twenty-eight participants took part in the experiment. Two participants were excluded because they had a negative correlation between their confidence and accuracy, indicating that they may not have given confidence ratings as instructed. Therefore, all analyses were based on the remaining 26 participants (15 females, age range = 18–22). All participants had normal or corrected-to-normal vision and provided informed consent. The experiment was approved by the Georgia Tech Institutional Review Board.

### Procedure

Participants indicated whether a grating of full contrast was drawn from one of two partially overlapping distributions. Distribution 1 tended to generate gratings with CCW orientations, whereas Distribution 2 tended to generate gratings with CW orientations. The experiment included two conditions. In the low variability condition, Distributions 1 and 2 were normal distributions, $N\left( {\mu ,{\sigma ^2}} \right)$, with means, $\mu $, of –2.4° and 2.4°, respectively, and a standard deviation (SD), $\sigma $, of 6° (Fig. 1). Note that here 0° indicates vertical orientation, negative numbers indicate CCW orientations, and positive numbers indicate CW orientations. In the high variability condition, Distributions 1 and 2 were simply scaled by a factor of 3 such that they were normal distributions with means of –7.2° and 7.2°, respectively, and SD of 18°. Therefore, the overlap between the two distributions (measured as the difference between their means divided by their SD) was identical for the two conditions and resulted in a maximum sensitivity of $d{^{^{\prime}}} = {{2.4 - \left( { - 2.4} \right)} \over 6} = {{7.2 - \left( { - 7.2} \right)} \over {18}} = 0.8$. Participants were explicitly given all of this information. Additionally, during the initial training, they were presented with a series of 25 randomly generated grating orientations from each of these four distributions in order to aid them in building their understanding of the task.

Each trial started with a fixation period (500 ms), followed by stimulus presentation (500 ms), untimed decision period, untimed confidence period, and a feedback screen presented for 500 ms (Fig. 1D). The grating (100% contrast, 5° diameter) was presented at fixation, and on each trial its orientation was randomly generated from Distribution 1 or 2. Confidence was provided on a 4-point scale where 1 indicates low confidence and 4 indicates high confidence. Participants selected the generating distribution using the left and right arrows on a computer keyboard with their right hand and gave a confidence rating using the 1–4 keys using their left hand. Trial-by-trial feedback was provided throughout the whole experiment.

The experiment was organized in four runs each consisting of four 50-trial blocks for a total of 800 trials. Each block consisted of only low or high variability trials and the condition was clearly indicated before the beginning of the block. The low and high variability blocks alternated with the identity of the first block randomly chosen for each participant. Successive blocks were separated by 15-s breaks, while successive runs were separated by self-paced breaks. Before the beginning of the experiment, participants were given four blocks of training with a total of 100 trials.

It should be emphasized that participants were informed about all aspects of the experimental design, as well as about the optimal strategy of keeping two separate sets of criteria for the low and high variability conditions. An alternative design option would have been not to inform participants about the existence of low and high variability conditions at all. This would have likely resulted in a complete collapse of the confidence criteria for the two conditions (an extreme form of criterion attraction) but such an effect would not have reflected a limitation of human decision-making but rather a rational response strategy. The current design where participants are informed about all aspects of the experiment gives participants all information needed for optimal responses and can thus reveal the inherent limitations of human decision-making.

Participants completed the experiment on a 21.5-inch iMac monitor in a dark room. The distance between the monitor and the participants was 60 cm. The stimuli were created in MATLAB using Psychtoolbox 3 (Brainard 1997).

### Analyses

For the main analyses, I computed the mean confidence and RT for each participant. Furthermore, to compare performance across participants, I computed the signal detection theory (SDT) measure $d^{\prime}$. To do so, I calculated the hit rate (HR) and false alarm rate (FAR) by treating Distribution 2 as the target. The value of $d^{\prime}$ was computed separately for each condition using the formula:

$$\begin{equation*}d^{\prime}= {\varPhi ^{ - 1}}(\textit{HR}) - {\varPhi ^{ - 1}}\left( {FAR} \right)\end{equation*}$$

where ${\varPhi ^{ - 1 }}$ is the inverse of the cumulative standard normal distribution that transforms HR and FAR into *z*-scores.

To assess the presence of criterion attraction, I computed the locations of the decision thresholds, ${t_i}$, expressed in stimulus space in each condition using SDT by applying the formula:

$$\begin{equation*}{t_i} = - {1 \over 2}\left( {{\varPhi ^{ - 1}}\left( {H{R_i}} \right) + {\varPhi ^{ - 1}}\left( {FA{R_i}} \right)} \right) \times \sigma \end{equation*}$$

where ${t_i}$, $H{R_i}$, and $FA{R_i}$ are the criterion location, hit rate, and false alarm rate for the ${i^{th}}$ criterion ($i = - 3, - 2, \ldots , 3$), and $\sigma $ is the standard deviation of the generating distributions. Note that ${t_0}$ is the criterion that separates decisions for Distribution 1 versus Distribution 2, while the confidence criteria $ \pm {t_k}$ for $k = \left\{ {1, 2, 3} \right\}$ separate the confidence ratings of $k$ and $k + 1$ (Macmillan and Creelman 2005). Negative criterion locations (i.e. $i \lt 0$) separate successive confidence ratings for ‘Distribution 1’ decisions, while positive criterion locations (i.e. $i \gt 0$) separate successive confidence ratings for ‘Distribution 2’ decisions. $H{R_i}$ was estimated such that for positive values of $i$, $H{R_i}$ is the proportion of trials where Distribution 2 was used to generate the stimulus and the participant chose Distribution 2 with a confidence rating higher than $i$. For negative values of $i$, $H{R_i}$ is the proportion of trials where Distribution 2 was used to generate the stimulus and the participant chose either Distribution 2 regardless of confidence or Distribution 1 with confidence rating lower than or equal to $ - i$. $FA{R_i}$ was computed equivalently but for trials where Distribution 1 was used to generate the stimulus.

To assess the presence of criterion attraction, I computed the ratio ${r_i} = {{{t_{i, HighVar}}} \over {{t_{i,LowVar}}}}$ for each pair of confidence criteria in the high and low variability conditions for each confidence criterion $i = - 3, - 2, - 1,1,2,3$. Since the stimulus distributions for the low and high variability conditions were identical except for scaling by a factor of 3, in the absence of internal noise, optimal confidence placement requires that the confidence criteria are also offset such that ${r_i} = 3$ for all $i$. On the other hand, criterion attraction would result in the confidence criteria being offset by a factor smaller than 3 (Fig. 1B). It should be noted that the optimal ratios ${r_i}$ also depend on the internal noise associated with the perception of the stimuli, which I did not measure. However, previous research suggests that the internal noise for orientation detection of Gabor patches of full contrast presented for a long period (500 ms in the current experiment) is very small and typically lower than 1° (Sally and Gurnsey 2004; Beaudot and Mullen 2006; Bang *et al.* 2019). This is further corroborated by the fact that the observed $d^{\prime}$ levels were very close to the maximum possible value of 0.8 (see Results). Importantly, internal noise of 1° would have an almost negligible effect on the optimal ratios ${r_i}$. Indeed, such internal noise would increase the SD of the high and Low variability conditions to $\sqrt {{{18}^2} + {1^2}} = 18.03$° and $\sqrt {{6^2} + {1^2}} = 6.08$° and would therefore result in a negligible reduction of the optimal ${r_i}$ from 3 to 2.964. Therefore, realistic levels of internal noise would have a very small effect on the optimal ratios ${r_i}$ and are thus not considered further here.

Because the stimulus orientation values were drawn randomly from the generating distributions, there were slight variations in the maximum accuracy for the two conditions across participants. To remove such variability, for each participant, I computed the number of congruent trials (where the stimulus orientation had the same polarity as the mean of the generating distribution) for each of the two conditions. If the low (high) variability condition had $m$ more congruent trials, then I excluded $m$ congruent and trials from the low (high) variability condition and $m$ incongruent trials from the high (low) variability condition. The process ensured that there were equal numbers of congruent and incongruent trials in the two conditions. The excluded trials were removed from the end of the experiment for each participant. On average, this procedure resulted in excluding 20 trials per participant for a 2.5% exclusion rate.

Statistical tests included standard frequentist tests such as *t*-tests and correlations. In addition, where appropriate, I also performed Bayesian tests to quantify the evidence for either the null or alternative hypotheses. Conventionally, the null hypothesis is considered supported for Bayes factor values of BF_01_ > 3, whereas the alternative hypothesis is considered supported for BF_10_ > 3.

### Model fitting

The main analyses above can show qualitatively the existence of criterion attraction but cannot be used to quantify its strength. Indeed, although the strength of the criterion attraction can be directly computed for each confidence criterion, there is no principled way in which these values can be combined to arrive at a single quantitative estimate per participant. For example, some participants have relatively few high-confidence responses, making the estimation of the extreme criteria (e.g. ${t_{ - 3}}$ and ${t_3}$) relatively noisy. Therefore, a simple formula, such as averaging the estimated criterion attraction values for all confidence criteria, is likely to result in an imprecise estimate of the true criterion attraction.

Therefore, to precisely quantify the degree of criterion attraction, I specified a simple process model of criterion attraction. Fitting the model to the raw data allowed me to arrive at the best quantitative estimate of the strength of criterion attraction. According to the model, participants start with a set of criteria, ${t_i}$, used in the low variability condition with $i = \left\{ { - 3, - 2, \ldots , 3} \right\}$. Optimally, if the criteria ${t_i}$ are used in the low variability condition, then the same criteria should be scaled by a factor of 3 and used in the high variability condition. However, in the presence of criterion attraction, the criteria in the low variability condition increase by a factor of $\alpha $ and become equal to $\alpha \times {t_i}$, while the criteria in the high variability condition decrease by a factor of $\alpha $ and become equal to ${{3 \times {t_i}} \over \alpha }$. Note that a value of $\alpha $ determines the strength of criterion attraction with $\alpha = 1$ corresponding to no criterion attraction and $\alpha = \sqrt 3 = 1.73$, corresponding to a situation where the two sets of criteria collapse onto a single set of identical values.

The model was fit to the data from each participant using the Bayesian Adaptive Direct Search toolbox, version 1.0.5 (Acerbi and Ma 2017). The model had seven free parameters: ${t_i}$ for $i = \left\{ { - 3, - 2, - 1, 1, 2, 3} \right\}$ and $\alpha $. For simplicity, ${t_0}$ was set to 0. The fitting was performed on the actual stimulus orientations encountered by each individual participant. To find the best fit, I computed the log-likelihood value associated with the full distribution of probabilities of each response type, as done previously (Yeon and Rahnev 2020; Shekhar and Rahnev 2021b):

$$\begin{equation*}Log\ likelihood = \mathop \sum \limits_{i,j,k} {\rm{log}}({p_{ijk}})*{n_{ijk}}\end{equation*}$$

where ${p_{ijk}}$ and ${n_{ijk}}$ are the response probability and the number of trials, respectively, associated with the Distribution $i \in \left\{ {1,2} \right\}$, confidence rating $j \in \left\{ {1,2,3,4} \right\}$, and condition $k$, where $k = 1$ corresponds to the low variability condition and $k = 2$ corresponds to the high variability condition. The best fit was determined as the set of parameters that maximized the log-likelihood value. Finally, I examined the resulting $\alpha $ values for each participant as the estimate of the strength of criterion attraction and performed *t*-tests to compare the resulting values to 1.

### Data, materials, and code

All data and codes for the analyses have been made freely available at https://osf.io/g32tv/. The repository also includes the codes used to collect the data and can be reused by anyone who may want to employ this method of generating confidence–accuracy dissociations. In addition, the data have been uploaded to the Confidence Database (Rahnev *et al.* 2020).

## Results

I investigated whether a robust confidence–accuracy dissociation could be achieved by exploiting the principle of criterion attraction in an external noise paradigm. Participants judged which of the two distributions generated a given stimulus and provided a confidence rating on a 4-point scale (Fig. 1D). Critically, two conditions—low variability and high variability (that were simply scaled versions of each other; Fig. 1A and B)—were presented in blocks of 50 trials. If the confidence criteria for the two conditions become attracted to each other, this would produce a confidence–accuracy dissociation.

### Low and high variability conditions exhibit robust confidence–accuracy dissociation

**Figure 2.**
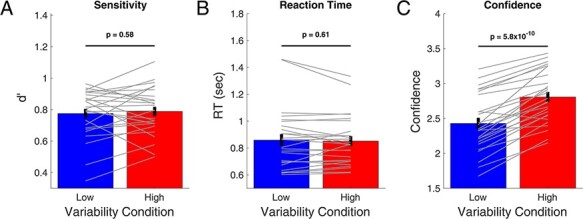
Confidence–accuracy dissociation between the low and high variability conditions. The low and high variability conditions were matched in terms of both $d^{\prime}$ (A) and RT (B), as also confirmed by a Bayes factors analysis. However, confidence was substantially higher in the high variability condition with the effect appearing in 25 of the 26 participants (C). Gray lines show individual participant data, and error bars show SEM

I first compared the sensitivity (as computed by the SDT measure $d^{\prime}$) and RT between the two conditions. I found that participants performed equally well in the low variability ($d^{\prime}= 0.78$) and high variability ($d^{\prime}= 0.79$) conditions [*t*(25) = 0.56, *P* = 0.58, Cohen’s *d* = 0.11, BF_01_ = 4.17; Fig. 2A]. Furthermore, the performance in each condition was indistinguishable from the maximum possible value of $d^{\prime}= 0.8$ [low variability condition: *t*(25) = –0.83, *P* = 0.4, Cohen’s *d* = –0.16, BF_01_ = 3.53; high variability condition: *t*(25) = –0.38, *P* = 0.7, Cohen’s *d* = –0.08, BF_01_ = 4.51], suggesting that the internal noise associated with the perception of the Gabor patches must have been minimal. Similar to $d^{\prime}$, RT was also equated between the low variability (mean RT = 859 ms) and high variability (mean RT = 852 ms) conditions [*t*(25) = –0.52, *P* = 0.61, Cohen’s *d* = –0.10, BF_01_ = 4.26; Fig. 2B]. These results show that the two conditions were very well matched in difficulty as measured both by stimulus sensitivity and RT.

Despite the close match in performance, there was a large difference in confidence between the two conditions (Fig. 2C). Specifically, the low variability condition resulted in much lower average confidence (2.43) than the high variability condition (2.81) with the difference exhibiting a very large effect size [*t*(25) = 9.7, P = 5.8 × 10^–10^, Cohen’s *d* = 1.9; BF_10_ = 1.9 × 10^7^]. Furthermore, the higher confidence in the high variability condition was present in 25 of the 26 participants (96%), demonstrating that the difference in confidence was extremely consistent across participants.

**Figure 3.**
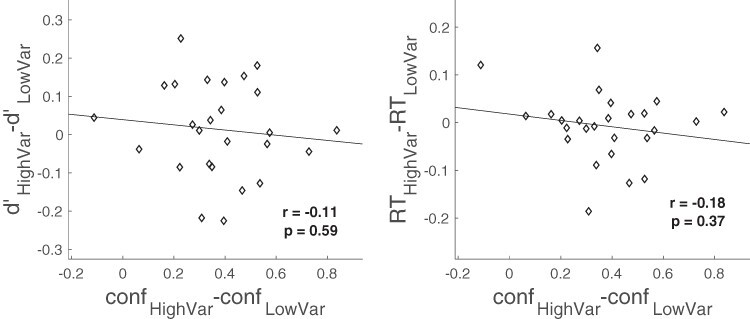
Confidence effects are not linked to ${\bf{\it{d^{\prime}}}}$ or RT effects. Individual differences in the confidence effect (difference in confidence in the high and low variability conditions) were not related to individual differences in the $d^{\prime}$ or RT effects (difference in $d^{\prime}$ or RT in the high and low variability conditions). Diamonds represent individual participants; the black line depicts the line of best fit

To further establish that the difference in confidence is independent of any effects on performance, I examined whether participants who tended to have larger confidence effects also exhibited a corresponding difference in $d^{\prime}$ or RT. I found that this was not the case. Specifically, participants who exhibited large confidence effects (i.e. large difference between the confidence in the high and low variability conditions, $con{f_{HighVar}} - con{f_{LowVar}}$) were not more likely to have a large difference in $d^{\prime}$ (correlation between $con{f_{HighVar}} - con{f_{LowVar}}$ and $d_{HighVar}^{^{\prime}} - d_{LowVar}^{^{\prime}}$: *r* = –0.11, *P* = 0.59; BF_01_ = 5.73) or in RT (correlation between $con{f_{HighVar}} - con{f_{LowVar}}$ and $R{T_{HighVar}} - R{T_{LowVar}}$: *r* = –0.18, *P* = 0.37; BF_01_ = 4.43; Fig. 3). Therefore, the difference in confidence between the low and high variability conditions was not driven by the objective performance of the participants.

### Time course of the effects

**Figure 4.**
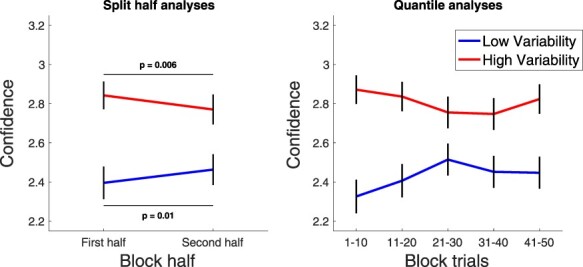
Time course of confidence effects. The difference in confidence between the low and high variability conditions was larger in the first than in the second half of the blocks. A finer split of each 50-trial block into five epochs shows that the difference in confidence is largest immediately at the start of a new block but remains relatively stable afterward, thus suggesting that the effects of criterion attraction can be relatively long-lasting

It is important to emphasize that the confidence–accuracy dissociation results above were obtained despite the fact that the low and high variability conditions were presented in separate blocks of 50 trials. Given this blocked design, one may expect that the criterion attraction would be larger at the beginning of each block when the influence of the previous block is likely to be the strongest. Indeed, I found that confidence increased from the first to the second half of the blocks for the low variability condition [first half = 2.40, second half = 2.46; *t*(25) = –2.80, *P* = 0.01, Cohen’s *d* = –0.55, BF_10_ = 4.80; Fig. 4, left] but decreased for the high variability condition [first half = 2.84, second half = 2.77; *t*(25) = 3.02, *P* = 0.006, Cohen’s *d* = 0.59, BF_10_ = 7.60]. Thus, the difference in confidence between the two conditions was significantly smaller in the second half of the blocks [first half difference = 0.45, second half difference = 0.31; *t*(25) = 4.07, *P* = 0.0004, Cohen’s *d* = 0.80, BF_10_ = 75.25]. These results demonstrate that the results were largest shortly after a switch from the previous block and suggest that these effects should be even larger in interleaved designs.

Analyzing each block in even finer intervals of 10 trials each revealed that this effect was driven mostly by a very large effect at the beginning of each block but without any evidence that the effect disappears by the end of the block (Fig. 4, right). Indeed, the difference in confidence between the two conditions was 0.54, 0.43, 0.24, 0.30, and 0.38, respectively, for the five sets of 10 trials constituting one 50-trial block. The smallest difference in confidence thus occurs toward the middle rather than at the end of the block. These results show that the criterion attraction effect is particularly strong at the very beginning of a block, but remains relatively stable afterward and does not disappear by the end of the block. The effects of criterion attraction thus appear relatively long-lasting and must extend for well over 50 trials.

### Confidence criterion locations

The results so far demonstrate that the low and high variability conditions give rise to a robust confidence–accuracy dissociation. To further examine the nature of this dissociation, I computed the location, ${t_i}$, of each decision criterion for both the low and high variability conditions in orientation space (see Methods).

As could be expected, the Criterion ${t_0}$ used to distinguish between Distributions 1 and 2 was very close to 0° (vertical) in both the low variability [${t_0} = 0.089$°, *t*(25) = 0.67, *P* = 0.51, Cohen’s *d* = 0.13, BF_01_ = 3.93] and the high variability conditions [${t_0} = 0.083$°, *t*(25) = 0.40, *P* = 0.69, Cohen’s *d* = 0.08, BF_01_ = 4.49], with no difference between the two conditions [*t*(25) = –0.04, *P* = 0.97, Cohen’s *d* = –0.01, BF_01_ = 4.82]. These results suggest that, as expected, participants made the primary decision by using a largely unbiased criterion centered on vertical orientation.

**Figure 5.**
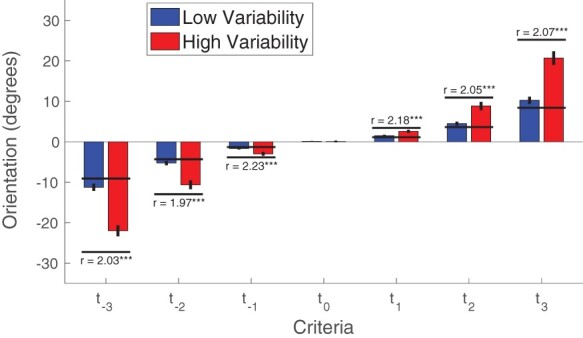
Criterion locations in the low and high variability conditions. The Criterion ${t_0}$ used to distinguish between Distributions 1 and 2 was not different between the two conditions. However, the ratio, $r$, between confidence criteria in the high and low variability conditions was significantly lower than 3 for all criteria. The black horizontal lines depict the expected criterion locations in the absence of criterion attraction. Error bars show SEM. *** *P* < 0.001

Critically, I examined the locations of the confidence criteria. In the absence of criterion attraction, the criterion locations in the high variability condition should be three times higher than in the low variability condition (Fig. 1B; note that this ratio could be expected to be slightly smaller due to internal noise; see Methods). However, I found the ratio, $r$, of their locations to be around 2 for all criteria (${r_{{t_{ - 3}}}} = 2.03$, ${r_{{t_{ - 2}}}} = 1.95$, ${r_{{t_{ - 1}}}} = 2.24$, ${r_{{t_1}}} = 2.19$, ${r_{{t_2}}} = 2.05$, ${r_{{t_3}}} = 2.07$; Fig. 5). Since the ratios $r$ are not normally distributed, I performed nonparametric signed rank tests that confirmed that all of these ratios were significantly lower than 3 (all *P* values < 0.0035). To avoid the issue of having to use non-parametric tests, I also compared the criteria in the high variability condition with the criteria in the low variability condition multiplied by a factor of 3. I found that the scaled criteria in the low variability condition were always more extreme (either more negative or more positive) than the criteria in the high variability condition [${t_{ - 3}}$: *t*(25) = 7.31, *P* = 1.2 × 10^–7^, Cohen’s *d* = 1.43, BF_10_ = 1.2*10^5^; ${t_{ - 2}}$: *t*(25) = 6.08, *P* = 2.4 × 10^–6^, Cohen’s *d* = 1.19, BF_10_ = 7.8 × 10^3^; ${t_{ - 1}}$: *t*(25) = 4.06, *P* = 0.0004, Cohen’s *d* = 0.80, BF_10_ = 70.6; ${t_1}$: *t*(25) = 4.41, *P* = 0.0002, Cohen’s *d* = 0.87, BF_10_ = 166.7; ${t_2}$: *t*(25) = 6.74, *P* = 4.6 × 10^–7^, Cohen’s *d* = 1.32, BF_10_ = 3.6 × 10^4^; ${t_3}$: *t*(25) = 6.86, *P* = 3.5 × 10^–7^, Cohen’s *d* = 1.34, BF_10_ = 4.6 × 10^4^], thus confirming the existence of substantial criterion attraction.

### Computational modeling

**Figure 6.**
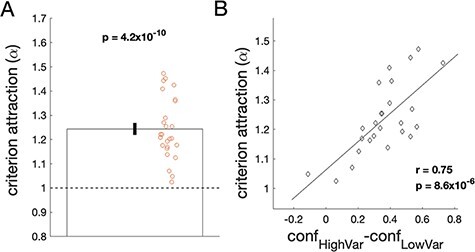
Quantifying the strength of criterion attraction. (A) Estimated criterion attraction $\alpha $. The dashed horizontal line depicts $\alpha = 1$ that corresponds to a lack of criterion attraction. Circles depict individual participants, and the error bar depicts SEM. On average, the criteria shifted by 24.4%, which is one-third of the shift required for the criteria in the two conditions to become identical. (B) Scatterplot of the estimated criterion attraction ($\alpha $) and the confidence effect ($con{f_{HighVar}} - con{f_{LowVar}}$) for each participant. The black line shows the line of best fit. The strong relationship between the two effects confirms that participants with high criterion attraction also had larger changes in confidence between the two conditions

Finally, I developed a simple computational model to more precisely quantify the strength of criterion attraction. The model assumed that the criteria in the low and high variability conditions are multiplicatively attracted to each other by a constant factor $\alpha $. Fitting the model to the data revealed that the criteria moved by an average factor of $\alpha = 1.244$ (range = 1.025–1.473, SD = 0.126), which was significantly larger than the factor of $\alpha = 1$ corresponding to a lack of criterion attraction [*t*(25) = 9.86, *P* = 4.2 × 10^–10^, Cohen’s *d* = 1.93, BF_10_ = 2.5 × 10^7^; Fig. 6A]. Note that if two criteria are originally separated by a factor of 3 (e.g. 10° and 30°), then after each of them is attracted to the other by a factor of 1.244 and they become separated by a factor of ${3 \over {{{1.244}^2}}} = 1.94$ (e.g. 12.4° and 24.1°). Importantly, the parameter $\alpha $ was estimated to be higher than 1 for all participants, suggesting that all 26 participants may have exhibited some level of criterion attraction.

As may be expected, the estimated strength of criterion attraction was strongly correlated with the confidence effect (across-subject correlation between $\alpha $ and $con{f_{HighVar}} - con{f_{LowVar}}$: *r* = 0.75, *P* = 8.6 × 10^–6^; Fig. 6B). Nevertheless, even though the criterion attraction was found for all participants and had a relatively large magnitude, the attraction fell far short of causing the same set of criteria to be used for both the low and high variability conditions. Indeed, a unique set of criteria across the two conditions would correspond to an $\alpha $ level of $\sqrt 3 = 1.732$ or a 73.2% change in the locations of the criteria. The observed average $\alpha = 1.244$ corresponds to a 24.4% change or exactly one-third of the strength of criterion attraction that would result in a unique set of criteria for the two conditions.

## Discussion

Many studies in the last decade have demonstrated the existence of confidence–accuracy dissociations (Wilimzig *et al.* 2008; Zylberberg *et al.* 2012, 2014, 2016; Vlassova *et al.* 2014; de Gardelle and Mamassian 2015; Koizumi *et al.* 2015; Rahnev *et al.* 2015; Song *et al.* 2015; Samaha *et al.* 2016; Spence *et al.* 2016, 2018; Boldt *et al.* 2017; Desender *et al.* 2018). Such findings have been foundational in discovering the mechanisms of confidence computations and have informed debates regarding the optimality of confidence ratings (Aitchison *et al.* 2015; Navajas *et al.* 2017; Rahnev and Denison 2018). However, most previous confidence–accuracy dissociations were of relatively small magnitude, were relatively inconsistent across participants, and included an RT confound. Here I developed a new experimental design based on external noise and the principle of criterion attraction. The design produced a confidence–accuracy dissociation of large magnitude and effect size, which was consistent across participants and free of RT confounds. These results establish a new method of inducing robust confidence–accuracy dissociations and have important implications about the ongoing debate regarding the malleability of subjective criteria.

### Mechanisms of criterion attraction

Criterion attraction is usually interpreted as a limitation of criterion setting where humans are unable to maintain two completely separate sets of criteria (Gorea and Sagi 2005). The idea is that the two sets of criteria pull on each other, thus leading to criterion attraction. In theory, criterion attraction could occur either at a perceptual or a post-perceptual (i.e. cognitive) level. In the current study, criterion attraction occurs at a post-perceptual level. Indeed, the perceptual stimuli here were very clear (presented at full contrast for a relatively long time) and therefore perception itself was unlikely to be substantially affected by the two conditions of the experiment (low vs. high variability conditions). Therefore, the observed confidence–accuracy dissociation in the present study is due not to differences in subjective experience but to cognitive limitations in converting the subjective experience into an appropriate confidence rating. As such, the present effect cannot be used in studies that seek to produce differences in subjective experience in the absence of performance confounds (Morales *et al.* 2015a, 2019). What the current paradigm produces instead is differences in reported confidence in the absence of performance confounds, with the confidence difference stemming from post-perceptual factors. This effect may still be useful for understanding subjective experience by providing a control case where a robust difference in reported confidence is not based on a change in subjective experience.

It is also important to clarify that criterion attraction is a separate phenomenon from other established effects such as regression to the mean. Regression to the mean occurs when in a random sequence of events an extreme observation is followed, with high probability, by a less extreme one. As such, regression to the mean is a purely statistical effect with no human participant necessary. On the other hand, criterion attraction is not a statistical necessity and instead reflects limitations of human criterion setting (e.g. it would be easy to design an artificial agent with no criterion attraction).

### Relationship to confidence–accuracy dissociation in previous research

As already reviewed in the Introduction, confidence–accuracy dissociations have been observed in many different studies using a variety of manipulations such as stimulus variability, attention, visual field location, and positive evidence bias. It is thus important to consider whether the current effects relate to any of these previous manipulations. For example, studies on stimulus variability (Zylberberg *et al.* 2014, 2016; de Gardelle and Mamassian 2015; Spence *et al.* 2016; Boldt *et al.* 2017) or positive evidence bias (Zylberberg *et al.* 2012; Koizumi *et al.* 2015) used stimulus manipulations in ways that may seem similar to the external noise manipulation here. However, it is important to clarify that none of these previous studies had a true external noise manipulation—in each case, there was a one-to-one mapping between the generating stimulus category and each stimulus. However, no such one-to-one mapping exists for external noise paradigms like in the present study; instead, a given stimulus feature (e.g. orientation) could be generated from either stimulus category. As such, the external noise manipulation used here is qualitatively different from the manipulations in most previous research on confidence–accuracy dissociations.

The presence of robust confidence differences in the absence of corresponding differences in either accuracy or RT may appear at odds with proposals that confidence is determined by the accuracy and RT of decision (Kiani *et al.* 2014; Zylberberg *et al.* 2016). However, these results only falsify an extreme version of this proposal where confidence is determined exclusively by accuracy and RT. Indeed, the current results show that confidence is influenced by factors separate from accuracy and RT, but cannot be used to argue that accuracy or RT are not causally important for confidence ratings in other designs. Similarly, criterion attraction can be modeled using any framework (e.g. signal detection and accumulation to bound) and thus cannot be used to distinguish between different frameworks of perceptual decision-making.

### Criterion attraction in previous research

Many studies by Gorea and Sagi suggested the presence of criterion attraction for various interleaved conditions (Gorea and Sagi 2000, 2001, 2002, 2005; Gorea *et al.* 2005; Zak *et al.* 2012). In some of these studies, the criteria even appeared to collapse onto the same unified criterion. Consequently, several more recent studies have simply assumed the presence of a unique criterion for different interleaved conditions without directly testing this assumption (Rahnev *et al.* 2011, 2012a,b; Solovey *et al.* 2015; Morales *et al.* 2015b; Li *et al.* 2018). These studies invariably used internal noise designs where criterion locations cannot be expressed in stimulus parameters and thus the presence of a unique criterion is perhaps impossible to test directly. However, this practice has recently been strongly criticized (Lee *et al.* 2021), especially in the light of a recent study using external noise where the criteria across different conditions were found not to be identical (Denison *et al.* 2018).

The notion of the confidence criteria attracting each other but without becoming identical may provide a unifying account for all studies to date. Findings that have been explained by a fully unified criterion across conditions could generally be explained just as well by criterion attraction (Morales *et al.* 2015b). On the other hand, the study by Denison *et al.* (2018) could also have featured criterion attraction that was simply too difficult to detect due to the study’s unique experimental design. Indeed, in that study, the optimal decision strategy in some conditions was not to give high confidence ratings at all, thus eliminating certain confidence criteria and making it difficult to test for criterion attraction. The possibility of an undetected criterion attraction in Denison *et al*.’s study is also supported by the fact that their data were best explained not by an optimal Bayesian model but by a heuristic one, which is consistent with the existence of decision biases.

These considerations suggest that the debate surrounding criterion interactions across conditions should perhaps move away from arguments of whether the criteria in different conditions are fully independent or fully unified. There already appears to be a substantial amount of evidence that the truth is somewhere in between. Therefore, future efforts would be better directed toward identifying the strength of criterion interactions and the factors that modulate this strength. By precisely quantifying the degree of criterion attraction in an external noise paradigm, the current paper makes a small step in this direction.

### Criterion attraction in the real world

All studies discussed here featured participants performing tasks on artificial stimuli in laboratory conditions. Therefore, an important question concerns whether similar criterion attraction exists in real-world settings. I think that it does. Indeed, virtually all perception occurs in conditions that vary from context to context and is therefore likely subject to criterion attraction. For example, when attempting to detect a mosquito, one should optimally adopt conservative criteria for plain backgrounds and liberal criteria for patterned backgrounds. While the relevant study has not been performed, I suspect that such situations are accompanied by strong criterion attraction where people have a much stronger bias to detect a mosquito against a plain background compared to a patterned one.

In addition, criterion attraction is likely to be relevant beyond perceptual tasks. Consider a teacher grading essays by fifth and eighth graders mixed in a single pile (but still clearly identified). Will the teacher be able to use age-appropriate grading criteria or will she exhibit criterion attraction and give relatively lower scores to the fifth graders and relatively higher scores to the eighth graders? Although I can only speculate, it seems likely that criterion attraction would occur in any situation where a person evaluates two or more groups of different abilities in mixed order, potentially with important societal consequences.

### Limitations

One limitation of the current study is that its design only points to the existence of criterion attraction but does not make it possible to measure the movement of the criteria in each condition independently. It is thus possible that, at least for some participants, the criteria from one condition remained constant and only the criteria in the other condition were attracted to the criteria in the first. I note that such an effect would still be a form of ‘criterion attraction’ and therefore would not change any of the conclusions of the current study. This question could be resolved by future studies that present each condition in isolation (ideally on separate days) before presenting them together in the same experimental session. Nevertheless, the split-half analyses already provide some evidence that both sets of criteria experienced attraction. Indeed, given that confidence increased from the first to the second half of blocks for the low variability condition but decreased from the first to the second half of blocks for the high variability condition, it appears that both sets of conditions experienced stronger criterion attraction in the first (compared to the second) half of blocks. Another limitation is that, as noted in the Methods, the current study did not measure the level of internal noise. Finally, the current study also leaves open the question of whether criterion attraction would have been even stronger in the absence of trial-by-trial feedback, or if the two conditions were interleaved in all blocks.

### Conclusion

I report on the strongest, to my knowledge, confidence–accuracy dissociation to date: even though both $d^{\prime}$ and RT were well matched across the two conditions (both BF_01_ > 3), confidence was robustly different. The effect was consistent across subjects and very strong in both magnitude (0.38-point difference on a scale where the extremes, 1 and 4, are 3 points apart) and effect size (Cohen’s *d* = 1.9). Therefore, the current study provides a robust method for inducing large confidence–accuracy dissociations.
